# Biosynthetic Pathway and Genes of Chitin/Chitosan-Like Bioflocculant in the Genus *Citrobacter*

**DOI:** 10.3390/polym10030237

**Published:** 2018-02-27

**Authors:** Masahiro Takeo, Kazuyuki Kimura, Shanmugam Mayilraj, Takuya Inoue, Shohei Tada, Kouki Miyamoto, Masami Kashiwa, Keishi Ikemoto, Priyanka Baranwal, Daiichiro Kato, Seiji Negoro

**Affiliations:** 1 Department of Applied Chemistry, Graduate School of Engineering, University of Hyogo, 2167 Shosha, Himeji, Hyogo 671-2280, Japan; takuya.i@eco-feed.org (T.I.); tada@bell-c.co.jp (S.T.); miyamoto-kouki@nikke.co.jp (K.M.); mashia@occn.zaq.ne.jp (M.K.); bambootail2016@gmail.com (K.I.); barnpriyanka@gmail.com (P.B.); negoro@eng.u-hyogo.ac.jp (S.N.); 2 Hyogo Analysis Center Co., Ltd., 4-10-8 Seimondori, Hirohata, Himeji, Hyogo 671-1116, Japan; kimura@hyobun.co.jp; 3 Microbial Type Culture Collection & Gene Bank (MTCC), CSIR-Institute of Microbial Technology, Sector 39-A, Chandigarh-160 036, India; mayil@imtech.res.in; 4 Department of Chemistry and Bioscience, Graduate School of Science and Engineering, Kagoshima 890-8580, Japan; kato@sci.kagoshima-u.ac.jp

**Keywords:** *Citrobacter*, biosynthesis, bioflocculant, chitosan, metabolic pathway

## Abstract

Chitin/chitosan, one of the most abundant polysaccharides in nature, is industrially produced as a powder or flake form from the exoskeletons of crustaceans such as crabs and shrimps. Intriguingly, many bacterial strains in the genus *Citrobacter* secrete a soluble chitin/chitosan-like polysaccharide into the culture medium during growth in acetate. Because this polysaccharide shows strong flocculation activity for suspended solids in water, it can be used as a bioflocculant (BF). The BF synthetic pathway of *C. freundii* IFO 13545 is expected from known bacterial metabolic pathways to be as follows: acetate is metabolized in the TCA cycle and the glyoxylate shunt via acetyl-CoA. Next, fructose 6-phosphate is generated from the intermediates of the TCA cycle through gluconeogenesis and enters into the hexosamine synthetic pathway to form UDP-*N*-acetylglucosamine, which is used as a direct precursor to extend the BF polysaccharide chain. We conducted the draft genome sequencing of IFO 13545 and identified all of the candidate genes corresponding to the enzymes in this pathway in the 5420-kb genome sequence. Disruption of the genes encoding acetyl-CoA synthetase and isocitrate lyase by homologous recombination resulted in little or no growth on acetate, indicating that the cell growth depends on acetate assimilation via the glyoxylate shunt. Disruption of the gene encoding glucosamine 6-phosphate synthase, a key enzyme for the hexosamine synthetic pathway, caused a significant decrease in flocculation activity, demonstrating that this pathway is primarily used for the BF biosynthesis. A gene cluster necessary for the polymerization and secretion of BF, named *bfpABCD*, was also identified for the first time. In addition, quantitative RT-PCR analysis of several key genes in the expected pathway was conducted to know their expression in acetate assimilation and BF biosynthesis. Based on the data obtained in this study, an overview of the BF synthetic pathway is discussed.

## 1. Introduction

Chitin/chitosan, a polysaccharide consisting of *N*-acetylglucosamine (GlcNAc) and/or glucosamine (GlcN) linked through β-1,4-glycosidic linkages, is one of the most abundant polysaccharides in nature [1,2]. Although chitin/chitosan is distributed in a wide range of living organisms [2,3], it is industrially produced from the exoskeletons of crustaceans such as crabs, shrimps, prawns, lobsters, and krill because waste from food-processing industry provides an abundant source of this material [2,3]. Due to its useful biological properties (e.g., biocompatibility, biodegradability, and antimicrobial activity) and chemical modification potentials through its reactive functional groups (–OH, –NH_2_, and –COOH) [2,4,5], chitin/chitosan has been used in a wide range of fields including biomedical, pharmaceutical, food production, and wastewater treatment fields [3,5,6]. However, time-consuming and costly extraction/purification processes (for the solubilization of chitin and the removal of proteins, minerals, and colors) are required for the production [3]. In addition, the supply of the waste depends on seasonal yields of the crustaceans and geographic conditions. Therefore, as an alternative, the production of chitin/chitosan from microbial sources has been considered and attempted [7]. In the case of microbial sources, the production can be conducted throughout the year using biotechnological processes, and the quality of the product is generally stable. The major source of such chitin/chitosan is fungal mycelia, because the cell walls of fungi contain large amounts of chitin, and fungal mycelia can be obtained as waste from mushroom production and from the fermentation industry [3,7]. However, the extraction/purification processes cannot be omitted, although they may be somewhat simpler than that from the waste of the crustaceans. If chitin/chitosan were secreted by microbial cultures in a soluble form, its production and the downstream processes for its production could be greatly simplified.

In 2000, Fujita et al. reported that the enterobacterial strain *Citrobacter* sp. TKF04, which produces a bioflocculant (BF) from acetate and propionate [8]. By the chemical analysis, the BF was found to be a high-molecular-weight (around 320 kDa) polysaccharide consisting of GlcNAc and GlcN, similar to chitin/chitosan [8]. Infrared spectroscopic analysis showed that it has a very similar structure to those of commercial chitin/chitosan products [8] and the deacetylation degree was estimated as 50–60%. Chitinase and chitosanase preferentially degraded it, resulting in significantly-reduced flocculation activities [8]. Later, Son et al. and Kim et al. also reported similar chitin/chitosan-like polysaccharide-producing enterobacterial strains, *Enterobacter* sp. BL-2 and *Citrobacter* sp. BL-4 [9,10]. Hence, we collected 36 *Citrobacter* strains from various microbial culture collection centers and measured their flocculation activities. We found that 21 strains belonging to four species (*C. freundii*, *C. braakii*, *C. youngae*, and *C. werkmanii*) showed flocculation activity when grown on acetate [11]. We confirmed that five selected strains with high flocculation activity secreted the chitin/chitosan-like BF with wide molecular weight distributions (with the peak tops of >1660 kDa in the gel filtration chromatography) into the culture medium [11]. These results demonstrate that many strains belonging to the genus *Citrobacter* have metabolic potential to produce the chitin/chitosan-like BF as a soluble form from acetate. We are very interested in the chitin/chitosan-like BF biosynthetic pathway.

**Figure 1:**
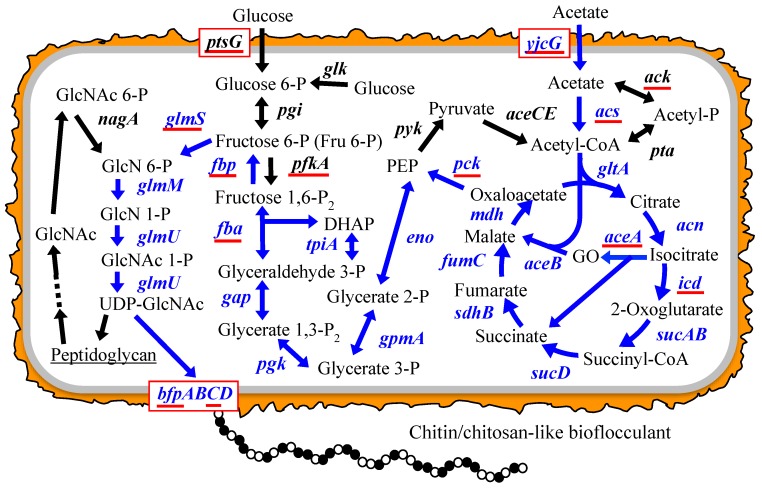
Proposed bioflocculant (BF) synthetic pathway and the genes of *Citrobacter freundii* IFO 13545 involved in the pathway. Blue arrows and gene names indicate the proposed BF synthetic pathway and the genes encoding the enzymes involved in this pathway. The genes underlined in red were used in a qRT-PCR study to determine the level of gene expression in glucose medium (GM) and in acetate medium (AM). Abbreviations: BF, bioflocculant; GO, glyoxylate; P, phosphate; P_2_, diphosphate; DHAP, dihyroxyacetone-P; PEP, phosphoenolpyruvate; GlcN, glucosamine; GlcNAc, *N*-acetylglucosamine.

Based on known bacterial acetate metabolic and peptidoglycan synthetic pathways [12,13], we expected the BF synthetic pathway of *Citrobacter* strains to be as follows (Figure 1): acetate is first converted into acetyl-CoA, which is further transformed into fructose 6-phosphate (Fru 6-P) through the TCA cycle, the glyoxylate shunt, and gluconeogenesis. Next, Fru 6-P enters into the hexosamine synthetic pathway (hereafter referred to as the hexosamine pathway) where it is transformed into UDP-*N*-acetylglucosamine (UDP-GlcNAc), which is finally used as a direct precursor for the extension of the BF polysaccharide chain. Unfortunately, little information is available on the polymerization and secretion of bacterial chitin/chitosan. Recently, we performed the draft genome sequencing of four *Citrobacter* strains, including *C. freundii* IFO 13545. We identified all the candidate genes corresponding to all the enzymes in the chitin/chitosan-like BF biosynthetic pathway in the 5420-kb genome sequence of IFO 13545 (Figure 1 and Table 1). To confirm the involvement of the products of these genes in acetate assimilation and BF biosynthesis, we conducted a gene disruption study involving several key genes of the pathway (Figure 1) in which we investigated the effects of the gene disruption of these genes on cell growth on acetate and on flocculation activity. In addition, through this gene disruption study, we identified a candidate gene cluster for the polymerization and secretion of the BF and report it here.

## 2. Materials and Methods 

### 2.1. Bacterial Strains, Media, and Culture Conditions

Two BF-producing strains, *C. freundii* IFO 13545 (Figure S1) and *C. freundii* GTC 09479 [11], were used in this study. *Escherichia coli* JM109 (Takara Bio, Kyoto, Japan) was also used as a host for the construction of recombinant DNA molecules. Acetate medium (AM) (10 g CH_3_COONa, 0.1 g yeast extract, 1.0 g (NH_4_)_2_SO_4_, 1.0 g K_2_HPO_4_, 0.05 g NaCl, 0.2 g MgSO_4_·7H_2_O, 0.05 g CaCl_2_, and 0.01 g FeCl_3_ in 1 L, pH 7.2) [11] and glucose medium (GM, to which glucose was added at 7.32 g·L^−1^ instead of acetate in AM to yield the same carbon content in both media) were used for the growth and BF production by *Citrobacter* strains, whereas LB medium [14] was used for the cell growth of pre-cultures and for plasmid preparation. Kanamycin (Nacalai Tesque, Kyoto, Japan) and tetracycline (Nacalai Tesque) were added to the growth medium at 25 mg·L^−1^ when necessary. The cultivation conditions were the same as those used in a previous study [11]. Briefly, twenty-five milliliters of each medium was added to 100-mL flasks that had been sterilized by autoclaving, and the medium was inoculated with each bacterial strain. Then, the flasks were shaken on a reciprocal shaker at 30 °C and 125 rpm. For the selection and growth of the *glmS* disruptant, LB agar plates containing kanamycin were used after 50 μL of 10 mM glucosamine 6-phosphate (GlcN 6-P) was spread on the agar surface. GlcN 6-P was added to the liquid medium to a final concentration of 1 mM.

### 2.2. Measurement of Flocculation Activity and Determination of Flocculation Titer

Flocculation activity was measured using kaolin suspensions as described previously [11]. A two-fold dilution series of the culture supernatant was prepared with distilled water and the flocculation activity of each sample was measured. The flocculation titer (Ft) was defined as the fold dilution that resulted in 50% flocculation activity according to plots of fold dilution vs flocculation activity; it was calculated using the two data points of the flocculation activities that were closest to the 50% flocculation activity.

### 2.3. Preparation of DNA Fragments by PCR for Gene Disruption

**Figure 2:**
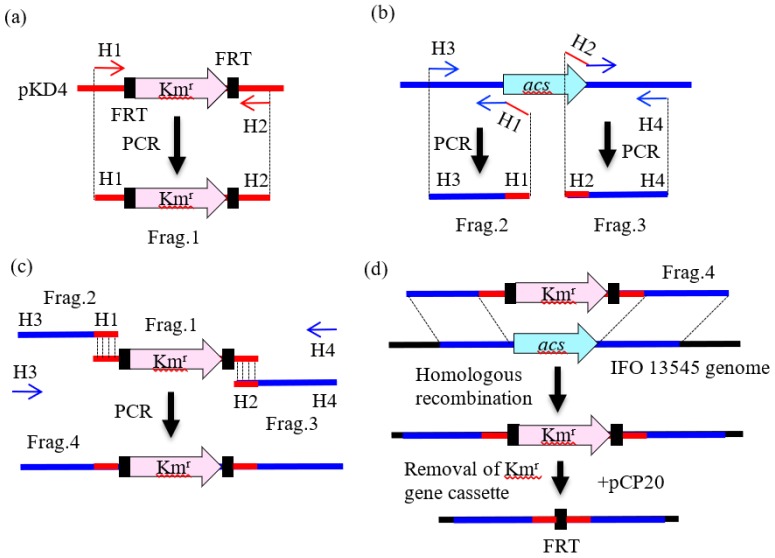
Construction of an acetyl-CoA synthetase gene-disruptant (*Δacs*) from *C. freundii* IFO 13545: (**a**) preparation of a Km^r^ gene cassette from pKD4 (Frag. 1); (**b**) amplification of the upstream and downstream regions of the target gene (*acs*) from the IFO 13545 genome (Frag. 2 and Frag. 3); (**c**) preparation of a DNA fragment for gene disruption (Frag. 4) from the amplified three fragments; and (**d**) disruption of the target gene (*acs*) by homologous recombination using Frag. 4. H1, H2, H3, and H4 indicate specific primers that were used or their sequence regions.

Total DNA was extracted from the cells of *Citrobacter* strains as described previously [11]. The primers used in this study for PCR are listed in Table S1. To disrupt genes in the *Citrobacter* strains, we employed a two-step PCR method based on the method of Yamamoto et al. [15]. As shown in Figure 2a, in the first step of this method, a kanamycin resistance gene cassette flanked by FLP recognition target (FRT) sequences was amplified from plasmid pKD4 [16] by PCR using the paired primers pKD4-Km-F and pKD4-Km-R. The 0.5-kb upstream and downstream flanking regions of the target gene to be disrupted were then amplified using primer pairs corresponding to both regions (Figure 2b). In the second step, a larger DNA fragment for homologous recombination was amplified by PCR using the outer primers (Figure 2c, H3 and H4) and the three PCR-amplified fragments as the templates (Figure 2c). The PCR mixture contained template DNA (50 ng), each primer (10 pmol), 4 μL of dNTP mixture (2.5 mM each), 5 μL of 10 × ExTaq buffer (Takara Bio), and 1.25 U of ExTaq polymerase (Takara Bio), and dH_2_O in 50 μL. The thermal program employed was as follows: initial denaturation at 94 °C for 5 min; 30 cycles of denaturation at 94 °C for 30 s, annealing at 55 °C for 30 s, and extension at 72 °C for 1.5 min for the first-step PCR (Figure 2a,b) or for 2.5 min for the second-step PCR (Figure 2c); and final extension at 72 °C for 5 min. In these reactions, the following primer pairs were used: acs-up-F and acs-up-R for the upstream region of *acs*; acs-down-F and acs-down-R for the downstream region of *acs*; aceA-up-F and aceA-up-R for the upstream region of *aceA*; aceA-down-F and aceA-down-R for the downstream region of *aceA*; glmS-up-F and glmS-up-R for the upstream region of *glmS*; glmS-down-F and glmS-down-R for the downstream region of *glmS*; cpsA-up-F and cpsA-up-R for the upstream region of *cpsA*; cpsA-down-F and cpsA-down-R for the downstream region of *cpsA*; bfpC-up-F and bfpC-up-R for the upstream region of *bfpC*; and bfpC-down-F and bfpC-down-R for the downstream region of *bfpC* (Table S1). For *nagA* disruption, a one-step PCR method was employed, in which nagA-Km-F with the 50-bp upstream flanking region of *nagA* at the 5′ end and nagA-Km-F with the 50-bp downstream flanking region of *nagA* at the 5′ end (Table S1) were used to amplify the Km^r^ gene cassette from pKD4. The resulting PCR fragment was used for homologous recombination after gel purification. The PCR products were analyzed by electrophoresis using 0.8% agarose gels (L03, Takara Bio).

### 2.4. Gene Disruption by Homologous Recombination

*Citrobacter* strains were first transformed with the Red helper plasmid pKD119 by electroporation (the conditions are described below). This plasmid promotes homologous recombination using the functions of the γ, β, and *exo* gene products of the λ phage [15,16,17]. Then, a selected transformant was grown at 30 °C in 100 mL of SOB medium [14] supplemented with tetracycline and l-arabinose (10 mM) (Nacalai Tesque). The cultured cells were harvested when the culture reached optical density at 600 nm (O.D._600_) of 0.6, washed three times with ice-cold 10% (*v/v*) glycerol, and resuspended in 10% glycerol solution at O.D._600_ = 12. The PCR products described in the previous section were purified by electrophoresis, and they were dissolved in a small amount of 10 mM Tris buffer (pH 8.0). The purified DNA fragments (10–100 ng) were electroporated into the cells (150 μL of the cell suspension) in a 0.1-cm gap cuvette using a Gene Pulser II (Bio-Rad Japan, Tokyo) under the following conditions: voltage, 2.25 kV; capacitance, 25 μF; resistance, 400 Ω. After electroporation, 1 mL of SOC medium (SOB medium containing 20 mM glucose) was added to the recovered cell suspension and the culture was incubated at 30 °C with agitation at 150 rpm for 1 h. Aliquots of the culture were spread on LB agar plates containing tetracycline and kanamycin, and the plates were incubated at 30 °C. The addition of kanamycin provided the basis for the first selection of the gene-disrupted strains (Figure 2d), and the addition of tetracycline ensured the maintenance of pKD119 in the cell. The kanamycin resistance gene cassette was removed from the genome of each strain using the FLP helper plasmid pCP20 (Figure 2d) [16] when necessary.

### 2.5. Southern Hybridization

**Figure 3:**
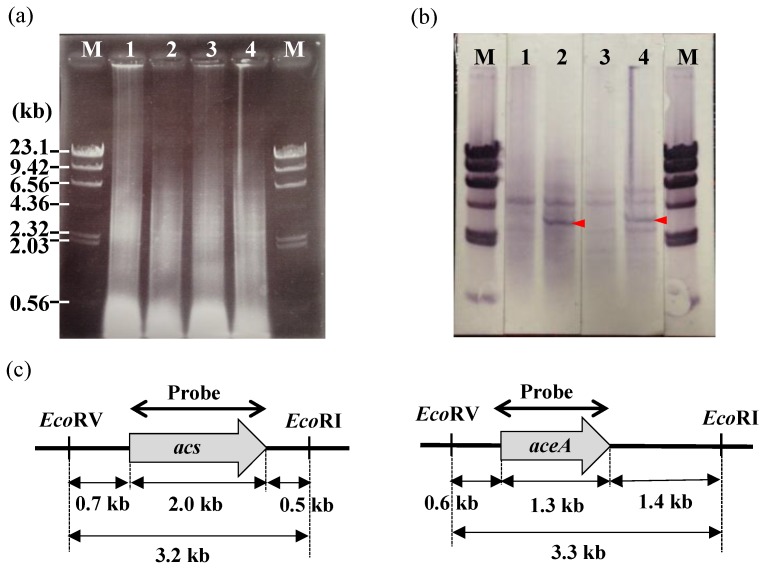
Electrophoresis of *Eco*RI-*Eco*RV-digested total DNA of *C. freundii* IFO 13545 and its gene-disrupted mutants, *Δacs* and *ΔaceA* (**a**); corresponding Southern blots (**b**); and restriction maps of the regions around *acs* and *aceA* in the IFO 13545 genome (**c**). Lane M: λDNA *Hin*dIII digest (size marker); Lane 1: *Δacs*; Lanes 2 and 4: IFO 13545 (wild type); Lane 3: *ΔaceA*. A probe based on the *acs* sequence was used in Lanes 1 and 2, whereas a different probe corresponding to the *aceA* sequence was used in Lanes 3 and 4. The arrow heads indicate the original gene fragments in the wild-type strain.

To confirm that the target gene was successfully replaced with the kanamycin resistance gene cassette (or successfully deleted), Southern hybridization was performed using a G-Capillary Blotter (TAITEC, Saitama, Japan), a Hybond-N+ membrane (GE Healthcare, Buckinghamshire, UK), and a DIG-High Prime DNA Labeling and Detection Kit (Roche Diagnostics, Mannheim, Germany) according to the manufacturer’s instructions [18]. The DNA regions used for the preparation of the probe are shown in Figure 3c for *acs* and *aceA*, in Figure S2c for *glmS*, and in Figure S3c for *bfpC*.

### 2.6. Batch Cultivation in a Mini-Jar Fermentor

Batch cultivation of *C. freundii* IFO 13545 was conducted in 500 ml of AM or GM using a mini-jar fermenter, NBS BioFlo115 (1.4-L vessel) (New Brunswick Scientific, Edison, NJ, USA) at 30 °C. During the cultivation, the pH value was automatically maintained below 8.5 in AM by addition of 0.5 M H_2_SO_4_ and above 7.2 in GM by addition of 1 M NaOH. The amount of dissolved oxygen was set at 20% of the saturated concentration and was automatically controlled by a combination of agitation (<150 rpm) and air supply (<2 vvm). The glucose and acetate in the culture were measured using enzyme assay kits (R-BIOPHARM AG, Darmstadt, Germany) according to the instructions provided with the kits.

### 2.7. Quantitative Reverse-Transcription-PCR (qRT-PCR)

During the abovementioned batch cultivation, cells were harvested by centrifugation (3000× *g*, 4 °C, 10 min) from 30 mL of each culture at three sampling points during the logarithmic growth phases. Total RNA was extracted from the cells using a PureLink^TM^ RNA Mini Kit (Life Technologies, Carlsbad, CA, USA) according to the manufacturer’s instructions. The RNA samples were digested with *Dpn*I (Takara Bio) under the conditions recommended by the supplier to remove any remaining host DNA (methylated DNA), followed by qRT-PCR analysis using a One Step SYBR^®^ PrimeScript™ RT-PCR Kit II (Takara Bio) and a Bio-Rad Real-Time PCR system MiniOpticon (Bio-Rad Japan, Tokyo, Japan). The following primers were used for cDNA synthesis and amplification of the target genes: aceA-RT-F and aceA-RT-R for *aceA*, acs-RT-F and acs-RT-R for *acs*, ack-RT-F and ack-RT-R for *ack*, bfpC-RT-F and bfpC-RT-R for *bfpC*, fba-RT-F and fba-RT-R for *fba*, fbp-RT-F and fbp-RT-R for *fbp*, glmS-RT-F and glmS-RT-R for *glmS*, icd-RT-F and icd-RT-R for *icd*, pck-RT-F and pck-RT-R for *pck*, pfkA-RT-F and pfkA-RT-R for *pfkA*, ptsG-RT-F and ptsG-RT-R for *ptsG*, yjcG-RT-F and yjcG-RT-R for *yjcG*, and 16S-RT-F and 16S-RT-R for the 16S rRNA gene (Table S1). These paired primers were designed to amplify an approximately 200-bp internal region of each target gene. To check the suitability of the paired primers for this analysis, amplification of the DNA fragments of the expected size from the total DNA of IFO 13545 was confirmed in advance by PCR using these paired primers (data not shown). A typical reaction mixture for qRT-PCR contained template RNA (50 ng), 2 × One Step SYBR^®^ RT-PCR Buffer 4 (Takara Bio) (12.5 µL), PrimeScript One Step Enzyme Mix 2 (Takara Bio) (1.0 µL), each primer (0.4 µM), and RNase-free dH_2_O (up to 25 µL). The thermal program employed was as follows: initial denaturation at 42 °C for 5 s, and at 95 °C for 10 s; 40 cycles of denaturation at 95 °C for 5 s, annealing and extension at 60 °C for 30 s; and a melting-curve step. Gene expression level was evaluated as the relative amount of specific RNA calculated from the qRT-PCR data using the 2^−ΔΔCT^ method [19].

### 2.8. Nucleotide Sequences Registered in the Databases

The genes involved in the acetate assimilation and BF synthesis in *C. freundii* IFO 13545 are listed in Table 1. The sequences of these genes have been registered in DDBJ/EMBL/GenBank under the accession numbers that are shown in the same table. 

## 3. Results

### 3.1. Putative BF Synthetic Pathway of C. freundii IFO 13545 and Its Related Genes

As described in the Introduction, based on known bacterial carbon metabolic pathways and on the peptidoglycan synthetic pathway [12,20], we expected the BF synthetic pathway of *C. freundii* IFO 13545 to be as follows (Figure 1): acetate is metabolized in the TCA cycle and the glyoxylate shunt via acetyl-CoA; Fru 6-P is generated from TCA cycle intermediates through gluconeogenesis; Fru 6-P enters into the hexosamine pathway and used to form UDP-GlcNAc, which serves as a direct precursor for extension of the BF polysaccharide chain. From the genome sequence of IFO 13545, we identified all the candidate genes corresponding to the enzymes that act during this metabolic pathway (Figure 1 and Table 1). These genes showed 79–98% nt sequence identity (90–100% aa sequence identities for the encoded proteins) with the corresponding genes of *Escherichia coli* K-12 MG1655 and with those of some other enteric bacterial strains (Table 1); the genome sequences of these strains were previously registered in the DNA databases, and the putative functions of the corresponding genes had been annotated. 

### 3.2. Effects of the Disruption of Acs or AceA on the Growth of IFO 13545 on Acetate

**Figure 4:**
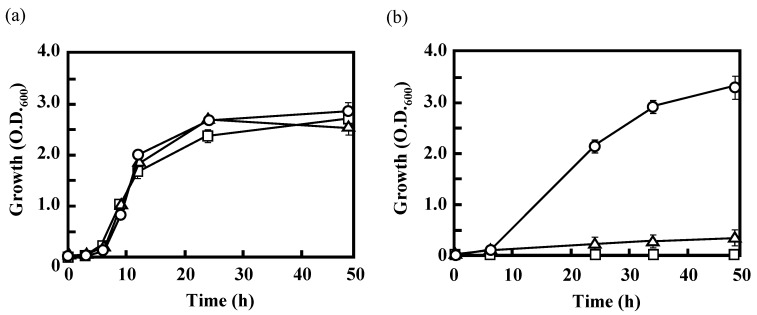
Growth of *C. freundii* IFO 13545 and its *acs* and *aceA* disruptants (*Δacs* and *ΔaceA*) on: glucose (**a**); and acetate (**b**). Symbols: circles, IFO 13545 (wild type); triangles, *Δacs*; squares, *ΔaceA*. This experiment was conducted in triplicate; the averages ± standard deviations of the values obtained are shown.

In *Escherichia coli*, there are two well-known major routes for the initial conversion of acetate into acetyl-CoA (Figure 1) [12,21]. In the first route, two enzymes, acetate kinase and phosphotransacetylase, catalyze two successive reactions that result in the conversion of acetate to acetyl-CoA via acetyl phosphate. The second route involves direct conversion of acetate into acetyl-CoA by acetyl-CoA synthetase. The reactions that occur in the first route are reversible, whereas the latter reaction is irreversible. To understand the first step in the acetate assimilation of IFO 13545, based on its genome sequence, a putative acetyl-CoA synthetase gene (*acs*, 1956 bp) was first disrupted by homologous recombination, and the gene disruption was confirmed by Southern hybridization. The Southern blotting revealed that the *acs* disruptant (*Δacs*) lacked a 3.2-kb *Eco*RV-*Eco*RI-digested DNA fragment, to which the *acs* probe hybridized in the wild-type strain (cf. Lanes 1 and 2 in Figure 3b). Although the disruptant displayed almost the same magnitude of growth on glucose as that of the wild-type strain, it showed little growth on acetate (Figure 4). This result suggests that IFO 13545 mainly uses the second route for acetate assimilation. Because *Δacs* was still able to grow very slowly on acetate, the first route may exist in this strain and may contribute slightly to acetate assimilation. In fact, two candidate genes (*ack* and *pta*) for acetate kinase and phosphotransacetylase were identified in the IFO 13545 genome sequence (Table 1) and the gene expression of *ack* was detected by qRT-PCR analysis as described later.

Next, if the acetyl-CoA that was formed enters into the TCA cycle and is used to make cell components and energy, the glyoxylate shunt should be used. The glyoxylate shunt includes two successive enzymatic reactions that catalyze the conversion of isocitrate to glyoxylate and succinate and then of glyoxylate and acetyl-CoA to l-malate (Figure 1). Passage through the shunt can shortcut two successive decarboxylation steps in the TCA cycle (from isocitrate to succinyl-CoA via 2–oxoglutarate), which release two carbon atoms as CO_2_ [12]. Therefore, using the glyoxylate shunt, the carbon atoms of acetate can be fixed to the cell. To confirm this assumption, a putative gene encoding isocitrate lyase (*aceA*, 1317 bp), which catalyzes the first reaction of the glyoxylate shunt, was disrupted in a manner similar to that described above. Southern hybridization demonstrated that the *aceA* disruptant (*ΔaceA*) lacked a 3.3-kb *Eco*RV-*Eco*RI-digested DNA fragment, to which the *aceA* probe hybridized in the wild-type strain (cf. Lanes 3 and 4 in Figure 3b). This disruptant, *ΔaceA,* no longer grew on acetate, although like the wild-type strain it grew well on glucose (Figure 4). This result suggests that the glyoxylate shunt mainly contributes to the acetate assimilation in IFO 13545.

### 3.3 Effects of the Disruption of GlmS and/or NagA on the Growth of IFO 13545 on Acetate and on Its Flocculation Activity

**Figure 5:**
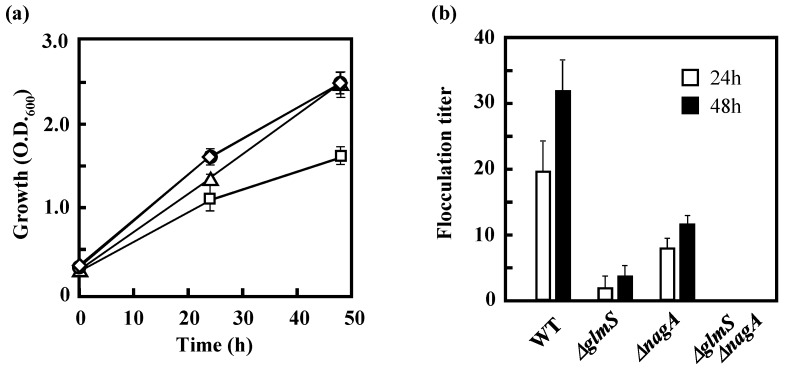
Growth of *C. freundii* IFO 13545 and its *glmS, nagA,* and *glmSnagA* disruptants (*ΔglmS*, *ΔnagA*, and *ΔglmSΔnagA*) on acetate (**a**); and their flocculation activities (**b**). Symbols: circles, IFO 13545 (wild type, WT); triangles, *ΔglmS*; diamonds, *ΔnagA*; squares, *ΔglmSΔnagA*. This experiment was conducted in triplicate; the averages ± standard deviations of the values obtained are shown.

**Figure 6:**
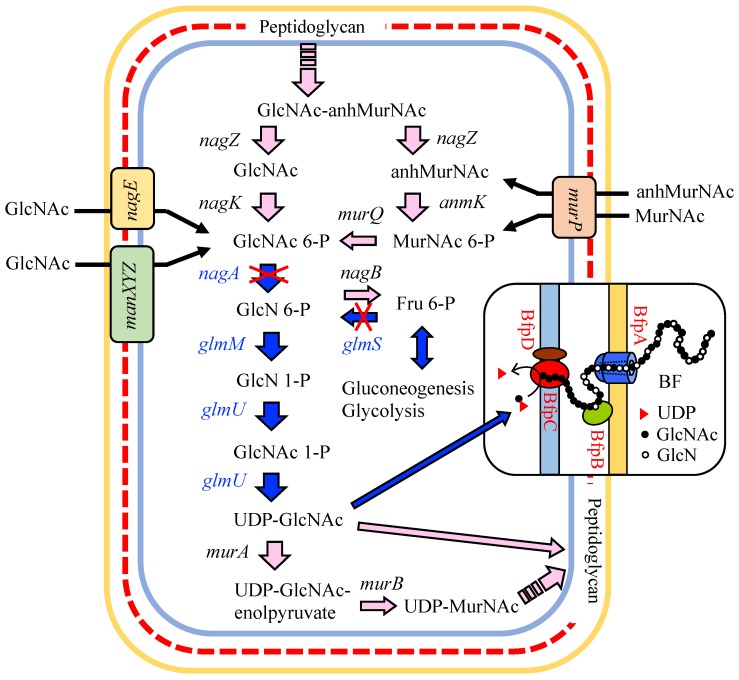
Amino sugar metabolism, peptidoglycan synthesis and recycling, and the putative polymerization and secretion mechanisms involved in production of the chitin/chitosan-like bioflocculant. These pathways and mechanisms are depicted based on information obtained from published reports [13,21,22,23]. Blue arrows and gene names indicate the proposed BF synthetic pathway and genes, respectively. Abbreviations: BF, bioflocculant; GlcN, glucosamine; GlcNAc, *N*-acetylglucosamine; MurNAc, *N*-acetylmuramic acid; P, phosphate; anh, anhydro.

Because the chitin/chitosan-like BF of IFO 13545 is composed of GlcNAc and/or GlcN [11], the precursor of the BF polymer was expected to be UDP-GlcNAc, which can be synthesized through the hexosamine pathway in the early stage of cell wall (peptidoglycan) biosynthesis (Figure 1) [13]. The starting compound of this pathway is Fru 6-P in glycolysis/gluconeogenesis, which can be formed from the intermediates of the TCA cycle through gluconeogenesis. In the first reaction of the hexosamine pathway, Fru 6-P is converted into GlcN 6-P by GlcN 6-P synthase (Figure 1). A putative gene encoding GlcN 6-P synthase (*glmS*, 1827 bp) was also identified in the IFO 13545 genome sequence and was found to be coupled with another hexosamine pathway gene, *glmU* (1368 bp), which encodes a bifunctional enzyme, GlcN 1-phosphate (GlcN 1-P) transacetylase/GlcNAc 1-phosphate (GlcNAc 1-P) uridyltransferase, in an operon (*glmUS*). To examine the effects of the disruption of *glmS* on cell growth on acetate and on flocculation activity, *glmS* was disrupted as described above. After homologous recombination, we attempted to isolate the *glmS* disruptant (*ΔglmS*) on LB agar plates containing kanamycin and tetracycline. However, no colonies were obtained in repeated experiments. We surmised that this disruption could cause a shortage of GlcN 6-P in the cell, resulting in severely decreased levels of UDP-GlcNAc, the common precursor for peptidoglycan and BF biosynthesis [13]. Thus, GlcN 6-P was added to the surfaces of the LB selection plates. This resulted in the appearance of several colonies on the plates used for selection of the transformants. Southern hybridization revealed that *glmS* had been successfully replaced with the kanamycin resistance gene cassette in these colonies (Figure S2). The growth of *ΔglmS* on acetate (in AM) was almost identical to that of the wild-type strain (Figure 5a). However, its flocculation activity was only 11.9% of that of the wild-type strain after 48 h-incubation (Figure 5b), indicating that *glmS* contributes significantly to BF biosynthesis. The remaining activity indicates the presence of alternative routes for supplying amino sugars for the BF biosynthesis. To identify the alternative routes, we decided to disrupt one possible route in which GlcNAc taken up from the outside of the cell and recycled from peptidoglycan is assimilated (Figure 6) [21,22]. The key step in the peptidoglycan recycling and the usage of external GlcNAc for BF synthesis is conversion of GlcNAc 6-P into GlcN 6-P by GlcNAc 6-P deacetylase, which is encoded by *nagA* (Figure 1 and Figure 6). Its candidate gene (1146 bp) was found in the IFO 13545 genome sequence (Table 1) and disrupted by homologous recombination. The *nagA* disruption was confirmed by PCR (data not shown). As shown in Figure 5a, the growth of the *nagA*-disruptant (*ΔnagA*) on acetate was almost the same as that of the wild-type strain, whereas its flocculation activity was reduced to 36.3% of that of the wild-type strain (Figure 5b). Thus, *nagA* disruption affects BF biosynthesis to a certain extent. We also constructed a double disruptant (*ΔglmSΔnagA*) by the same procedure to determine the simultaneous effect of the disruption of both genes on flocculation activity and on growth on acetate. The double disruptant completely lost flocculation activity (Figure 5b). This result indicates that the disruption of both genes almost completely blocked the supply of amino sugars for BF biosynthesis. However, the double disruptant was still able to grow on acetate (Figure 5a), suggesting the possibility of the existence of other amino sugar supply resources for the growth.

**Figure 7:**
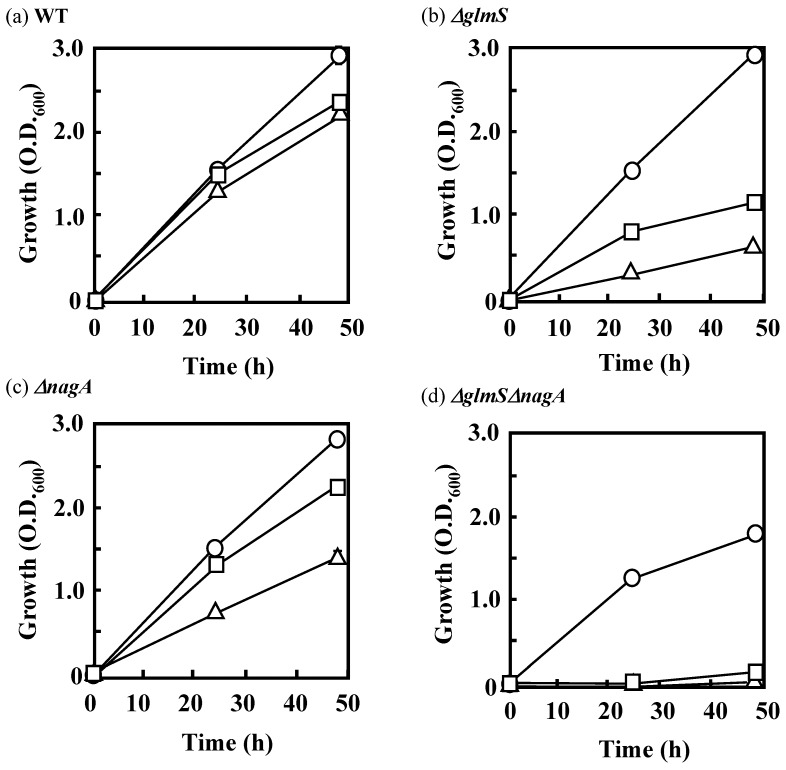
Growth of *C. freundii* IFO 13545 (wild type, WT) (**a**) and of its *glmS*, *nagA*, and *glmSnagA* disruptants (*ΔglmS* (**b**); *ΔnagA* (**c**); and *ΔglmSΔnagA* (**d**)) in acetate medium containing 0.1 g·L^−1^ yeast extract (circles), in acetate medium lacking yeast extract (triangles), and in acetate medium lacking yeast extract and supplemented with 0.2 g·L^−1^ GlcNAc (squares). This experiment was performed in triplicate; the averages ± standard deviations of the values obtained are shown. Almost all the standard deviations were within the symbol sizes.

To investigate the effects of the yeast extract used in AM as a supply of amino sugars from outside the cell, these *glmS* and/or *nagA* disruptants were cultivated in AM lacking yeast extract. Under these conditions, the wild-type strain showed growth almost identical to its growth in AM (Figure 7a), whereas the single disruptants, *ΔglmS* and *ΔnagA*, showed considerably slower growth than they displayed in the presence of yeast extract (Figure 7b,c). The double disruptant failed to grow in the absence of yeast extract (Figure 7d), suggesting that yeast extract, despite its presence at a low concentration (0.1 g·L^−1^ in AM), provides important factors necessary for the growth of the disruptants. Addition of GlcNAc at 0.2 g·L^−1^ to AM lacking yeast extract greatly improved the growth of *ΔglmS* and *ΔnagA* (Figure 7b,c), demonstrating that one of the necessary factors was GlcNAc. However, the double disruptant showed little growth on acetate even after the addition of GlcNAc (Figure 7d). The fact that the double disruptant was able to grow in the presence of yeast extract indicates that yeast extract contains other compounds that support the cell growth (probably other sugars that can be used in peptidoglycan synthesis). 

### 3.4. Identification of Genes that Are Involved in the Polymerization and Secretion of the Chitin/Chitosan-Like BF

In this gene disruption study, we attempted to identify genes that are involved in the polymerization and secretion of the chitin/chitosan-like BF, indispensable processes for BF production. However, limited information about these genes is currently available. From the IFO 13545 genome sequence, several genes that potentially encode enzymes involved in polysaccharide polymerization or membrane channel proteins involved in polysaccharide secretion were selected and independently disrupted by homologous recombination. In this way, we identified two intriguing genes, *cpsA* (1413 bp) and *bfpC* (1332 bp), whose disruption significantly affected the flocculating activity of IFO 13545. The aa sequence of the *cpsA* gene product exhibited 99–100% aa sequence identity with the undecaprenyl phosphate glucose phosphotransferases of several *Citrobacter* and *E. coli* strains (e.g., accession Nos. EKS56046, EOQ22059, and KEL78484), which catalyze the addition of the first hexose to undecaprenyl phosphate (a lipid carrier) that is anchored at the cell membrane for the biosynthesis of lipopolysaccharides (LPS) and capsular polysaccharides (CPS) [24,25]. When *cpsA* was disrupted by homologous recombination, the flocculation activity of the *cpsA* disruptant (*ΔcpsA*) was reduced to 62% of that of the wild-type strain (data not shown), although it grew considerably better on acetate than did the wild-type strain. 

The *bfpC* gene product exhibited 96–100% aa sequence identity with the putative glycosyltransferases (polysaccharide *N*-glycosyltransferases) of several *Citrobacter* and *E. coli* strains that are involved in the biosynthesis of poly-β-1,6-*N*-acetyl-d-glucosamine (PGA or PNAG) (e.g., accession nos. EEH94348, EHL86151, and KEL79567). PGA is required for the structural development and integrity of biofilms in a wide variety of Gram-positive and Gram-negative bacteria, and it has profound effects on host–microbe interactions [26,27]. A *bfpC*-disruptant (*ΔbfpC*) was also constructed from IFO 13545 by homologous recombination (Figure S3). It showed no flocculation activity. This result strongly suggests that *bfpC* encodes a glycosyltransferase that is required for the BF polymerization. To confirm the importance of *bfpC* in the BF biosynthesis, a *bfpC* homolog in a different BF-producing strain, *C. freundii* GTC 09479 (99.4% identity with the *bfpC* of IFO 13545, nt355104-nt356435 in accession No. AOMS01000025) was also disrupted by homologous recombination. The GTC 09479 disruptant also showed no flocculation activity. Accordingly, we concluded that this gene is indispensable for the BF biosynthesis in IFO 13545. Based on the sequence analysis, the *bfpC* gene product (BfpC) was determined to belong to the nucleotide-diphospho-sugar glycosyltransferase family 2 [28]; five aa residues, Asp^163^, Asp^256^, Gln^292^, Arg^295^, and Trp^296^, which are thought to be the catalytic residues [29], are conserved in this protein.

### 3.5. Expression of Key Genes Involved in Acetate Assimilation and BF Production in IFO 13545 

**Figure 8:**
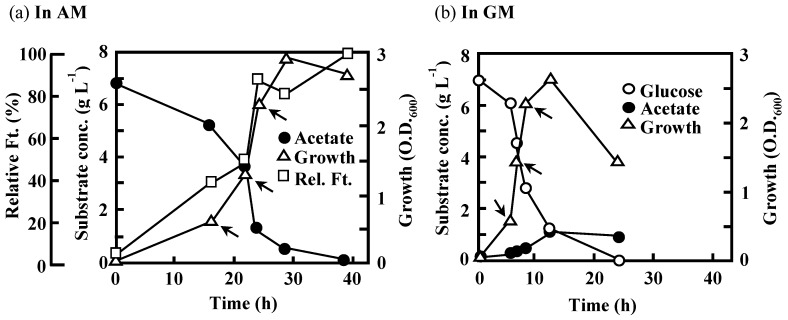
Changes in substrate concentration, growth, and relative flocculation activity (flocculation titer, Ft) during the batch cultivation of *C. freundii* IFO 13545 in: AM (**a**); and GM (**b**). Arrows indicate the sampling points of the cultures for qRT-PCR analysis (early-, mid-, and late-log phases in Figure 9).

**Figure 9:**
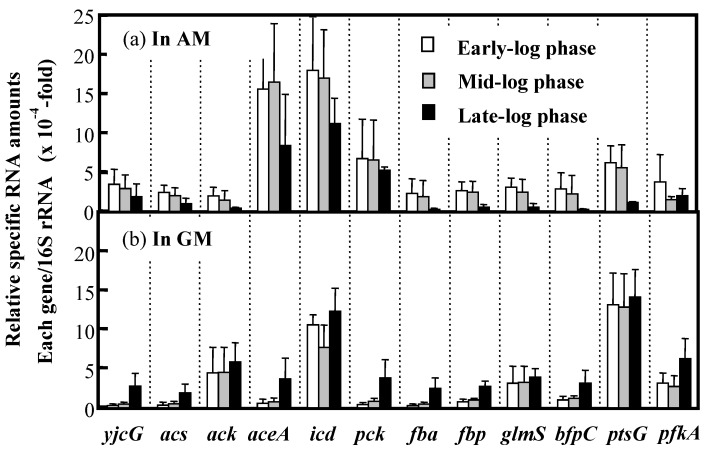
Relative gene expressions in the cells of *C. freundii* IFO 13545 grown in AM (on acetate) (**a**) or GM (on glucose) (**b**) compared to that of the 16S rRNA gene. The cells were grown in AM or GM and harvested at O.D._600_ = 0.52, 1.15, and 2.00 (early-, mid- or late-log phase, respectively). RNA samples were extracted from the cells and qRT-PCR was conducted using specific paired primers for each gene (Table 1). The average ± the standard deviation of the values obtained in three repeated experiments is shown. The relative amounts of RNA from the 16S rRNA gene in AM were 3.27 × 10^−2^ (early), 2.63 × 10^−2^ (mid), and 3.88 × 10^−2^ (late), whereas the amounts in GM were 3.39 × 10^−2^ (early), 2.11 × 10^−2^ (mid), and 3.35 × 10^−2^ (late).

To determine the expression levels of the abovementioned genes in IFO 13545 cells grown on acetate or glucose, qRT-PCR analysis was conducted using RNA samples obtained from cells grown in AM or GM. The RNA samples were prepared from IFO 13545 cells sampled at the early, middle, and late stages of logarithmic growth during batch cultivation in a mini-jar fermenter (Figure 8). The genes studied in this analysis were as follows: *yjcG*, *acs*, and *ack*, which encode proteins involved in the uptake of acetate and its initial conversion into acetyl-CoA; *aceA* and *icd*, which encode enzymes that act at the branching point in the TCA cycle for the glyoxylate shunt; *pck*, *fba*, and *fbp*, which encode proteins involved in gluconeogenesis; *ptsG*, *pfkA, and fba*, which are necessary for glucose uptake and glycolysis; and *glmS* and *bfpC*, which are required for the hexosamine pathway and BF polymerization, respectively (Table 1). Figure 9 shows the profile of gene expression normalized to the level of expression of the 16S rRNA gene. The expression levels of *yjcG*, *acs*, and *aceA* were higher in cells grown in acetate than in cells grown in glucose at the early- and mid-log phases. The expression of *aceA* was especially prominent, indicating its strong induction by acetate. This result is in good agreement with the result from the growth experiments using *Δacs* and *ΔaceA* (Figure 4): the disruption of these genes negatively affected the growth of the cells on acetate, resulting in no or little growth. The gene expression level of *icd* was also high in the acetate-grown cells as well as in the glucose-grown cells. The contribution of *aceA* and *icd* to carbon metabolism at the branching point for the glyoxylate shunt is discussed later in this work. It is worth noting that an increased level of *pck* expression for gluconeogenesis was detected in the acetate-grown cells. In addition, the expression of *fba*, and *fbp* was also higher during the early- and mid-log phases of growth, indicating that gluconeogenesis was promoted under conditions of acetate assimilation. The expression of *bfpC* was somewhat higher in acetate-grown cells than that in glucose-grown cells, whereas the expression of *glmS* was similar under the two growth conditions. At the late-log phase, the expression of all of the studied genes in acetate-grown cells declined, likely due to exhaustion of the carbon source (acetate) (Figure 8a). 

In contrast to the results for cells grown in acetate, in the glucose-grown cells, the gene expression levels of *ptsG* and *ack*, which encode a glucose-specific transporter and acetate kinase, respectively, were very high. Acetate kinase, the *ack* gene product, is known to produce acetate from acetyl-CoA during the overflow metabolism (fast unbalanced metabolism) of glucose in *E. coli,* whereas acetyl-CoA synthetase scavenges the accumulated acetate [30,31,32]. Notably, at the late-log phase, a low level of acetate formed from glucose had accumulated (Figure 8b); at that point, a metabolic adaptation from glucose to acetate (as carbon source) could have occurred because the expression levels of *yjcG*, *acs*, *aceA*, *pck*, *fba*, *fbp* and *bfpC*, which showed higher expression in the acetate-grown cells, increased at the late-log phase in the glucose-grown cells (Figure 9).

## 4. Discussion

In this study, to identify the BF synthetic pathway and the genes associated with this pathway in *C. freundii* IFO 13545, several key genes from the expected pathway were independently disrupted by homologous recombination. The poor growth of *Δacs* on acetate (Figure 4b) suggested that acetate is mainly converted into acetyl-CoA by acetyl-CoA synthetase (Acs). The higher gene expression level of *acs* observed in the acetate-grown cells compared to the glucose-grown cells (Figure 9) also supports this hypothesis. The acetyl-CoA that is formed from acetate can be easily metabolized in the TCA cycle. At the branching point in the TCA cycle for the glyoxylate shunt, isocitrate lyase (ICL, the *aceA* gene product) and isocitrate dehydrogenase (IDH, the *icd* gene product) compete for the same substrate, isocitrate (Figure 1). The level of expression of *aceA* in the acetate-grown cells was very high, and expression of this gene appeared to be strongly induced by acetate, as indicated by the gene expression study (Figure 9). Unexpectedly, the expression level of *icd* was also high in the acetate-grown cells. Thus, in the presence of acetate, both enzymes may be present in large amounts in the same cell. In *E. coli*, ICL has a much lower affinity for isocitrate (*K*_m_ = 604 μM) than IDH (*K*_m_ = 8 μM) [12]. If this is the case, major carbon flux tends to flow into the TCA cycle side (to 2-oxoglutarate) at the branching point. However, in *E. coli*, the activity of IDH is regulated by its reversible phosphorylation by IDH kinase/phosphatase, which is encoded by *aceK* in the *aceBAK* operon [12]. This operon was also detected in IFO 13545 (data not shown). Concomitant-up-regulation of *aceA* and *aceK* by acetate (>10-fold compared to by glucose) has been reported in the gene expression profiling studies of acetate-grown *E. coli* using DNA microarrays [33]. Therefore, in cells grown on acetate, IDH is always inactivated by phosphorylation even if a large amount of IDH protein exists in the cell, and most carbon flux flows into the glyoxylate shunt. Based on the data, we obtained on the growth of *ΔaceA* on acetate (Figure 4) and on the gene expression of *aceA* (Figure 9), this may occur in the IFO 13545 strain. 

Our qRT-PCR analysis of *pck*, *fbp*, and *fba* expression revealed that gluconeogenesis is greatly promoted in the acetate-grown cells. In addition, the disruption of *glmS* caused a noticeable decrease in flocculation activity, suggesting that the major BF synthetic pathway involves gluconeogenesis and the hexosamine pathway. Kim et al. conducted proteomic analysis of acetate metabolism in the polyglucosamine (PGB-2)-producing strain *Citrobacter* sp. BL-4 and found that 124 proteins were produced only in the acetate metabolism; these proteins included Acs, which acts during acetyl-CoA biosynthesis, and ICL, which acts in the glyoxylate shunt [34]. This result is reasonable and in good agreement with our results. Those authors also detected GlcN 6-P synthase (the *glmS* gene product, GlmS), which functions in the hexosamine pathway. However, in IFO 13545, the gene expression level of *glmS* in acetate-grown cells was relatively low compared to that in glucose-grown cells. One possible explanation for this is that amino sugar formation by GlmS is always necessary for peptidoglycan synthesis irrespective of the carbon source. However, the reaction catalyzed by GlmS may be a limiting step for BF biosynthesis by IFO 13545. Son et al. hypothesized that the genes associated with the hexosamine pathway (*glmU*, *glmS*, and *glmM*) are essential for the production of microbial polyglucosamine PGB-1 in *Enterobacter* sp. BL-2, and they introduced additional *glmS* copies (as a pBBR1MCS-2-based plasmid) into the BL-2 strain by conjugation to strengthen the pathway [9,35]. The resulting BL-2S strain produced a 1.5-fold more PGB-1 compared than the wild-type BL-2 strain. This strategy has been used to enhance the production of GlcNAc/GlcN by recombinant *E. coli* strains [36,37]. Therefore, this strategy would be a promising way to improve BF production by IFO 13545. In addition to *glmS*, we showed that *nagA* is also very important for growth and BF biosynthesis in IFO 13545 because it enables the cells to utilize amino sugars from outside the cell, and probably from the peptidoglycan recycling. By disruption of *nagA* and *glmS*, we showed that yeast extract can act as an amino sugar supply source in these disruption mutants. Based on the above results, the overview of the BF synthetic pathway from acetate (acetate assimilation, gluconeogenesis, and hexosamine pathway) was confirmed, although it will be important to complete the biochemical characterization of each enzyme step according to this pathway.

In this study, we searched for the genes involved in the polymerization and secretion of BF in the genome of IFO 13545. Currently, two major bacterial CPS/EPS biosynthesis systems, the Wzy- and ABC transporter-dependent CPS/EPS biosynthesis systems, are recognized [25]. We identified many gene clusters for ABC transporters in the IFO 13545 genome; however, based on sequence homology, those genes appeared to encode proteins that function in the transportation of amino acids and low-molecular weight ions from the sequence homologies (data not shown). We also found a gene cluster encoding a typical Wzy-dependent polysaccharide synthesis system that occupies over 20 kb in the genome; this cluster is very similar to the gene cluster that encode the enzymes involved in colanic acid biosynthesis in *E. coli* [38]. Some of the genes in the gene cluster encoding a Wzx homolog (flippase) and a Wza homolog (outer membrane channel protein) were independently disrupted, but this did not affect the flocculation activity of the disruptants; it was similar to that of the wild-type strain. We then identified *bfpC*, which is clustered with three other genes, named *bfpA* (2460 bp), *bfpB* (2019 bp), and *bfpD* (504 bp) as *bfpABCD*. These genes are transcribed in the same direction in that order. *bfpA* and *bfpB* are separated by a 12-bp gap and the stop codon of *bfpB* overlaps with the start codon of *bfpC*. Although *bfpD* lacks a typical start codon ATG coding for methionine, its tentative start codon, GTG, also overlaps with the stop codon of *bfpC*. These observations indicate that *bfpABCD* forms a single transcriptional unit (an operon). The *bfpABCD* gene cluster is similar to the *pgaABCD* gene cluster of *Escherichia coli* K-12 MG1655, which is involved in the biosynthesis and secretion of poly-β-1,6-*N*-acetyl-D-glucosamine (PGA) [23,39], although the sequence identities of the gene products encoded by *bfpABCD* and *pgaABCD* are relatively low. The *bfpABCD* gene products showed 44.5%, 52.3%, 67.2%, and 26.2% aa sequence identities with the corresponding *pgaABCD* gene products. The predicted functions of the *pgaABCD* gene products are as follows: PgaA is an 807-aa outer membrane protein that functions in the translocation of PGA; PgaB is a 672-aa polysaccharide *N*-deacetylase that is required for PGA deacetylation; PgaC is a 441-aa *N*-glycosyltransferase that is required for PGA polymerization; and PgaD is a 137-aa inner membrane protein that likely functions as a helper protein for PgaC [23]. Based on the available information on the functions of the *pgaABCD* gene products, we expected that the mechanisms of the polymerization and secretion of the chitin/chitosan-like BF are as shown in Figure 6. We performed the disruption of *bfpA*, *bfpB*, and *bfpD* in the same manner as for *bfpC*. All of these disruptions resulted in complete loss of the flocculation activity in IFO 13545 (data not shown). In addition, a trial, in which *bfpABCD* was introduced into the *bfpC* disruptant via an *E. coli* expression vector, resulted in the recovery of the flocculation activity (data not shown). Therefore, the *bfpABCD* genes are likely to encode proteins necessary for the polymerization and secretion of BF. As described above, Kim et al. conducted a proteomic analysis of the PGB-2-producing strain *Citrobacter* sp. BL-4 [34]. However, those authors did not identify *pgaABCD*- and *bfpABCD*-related gene products as acetate-induced proteins. Our qRT-PCR analysis indicated that the gene expression level of *bfpC* is not so high (Figure 9). Therefore, this gene cluster would not be found out without this gene disruption study.

## Figures and Tables

**Table 1:** Accession numbers of the registered genes in *Citrobacter freundii* IFO 13545.

Accession No.	Gene	Enzyme	Strain with Homologous Gene	Homologous Gene Accession No.	Identity at nt Sequence Level (%)	Identity at aa Sequence Level (%)
AB823554	*aceA*	isocitrate lyase	*E. coli* MG1655	U00096	86	96
AB823555	*aceB*	malate synthase	*E. coli* MG1655	U00096	84	92
AB823558	*acn*	aconitate hydratase I	*E. coli* MG1655	U00096	87	96
AB823559	*acs*	acetyl-CoA synthetase	*E. coli* MG1655	U00096	85	94
LC020545	*ack*	acetate kinase	*E. coli* MG1655	U00096	91	96
AB823580	*bfpA*	PGA export porin	*C. werkmanii*	KF057877	91	98
AB823581	*bfpB*	PGA *N*-deacetylase	*C. freundii* CFNIH1	CP007557	90	97
AB823582	*bfpC*	PGA synthase	*C. werkmanii* strain BF-6	KF057878	94	99
AB823583	*bfpD*	PGA biosynthesis protein	*C. freundii* CFNIH1	CP007557	88	95
LC020058	*cpsA*	undecaprenyl-phosphate glucose phosphotransferase	*C. freundii* MTCC1658	CP007557	86	100
AB823561	*eno*	enolase	*E. coli* MG1655	U00096	93	97
AB823562	*fba*	fructose bisphosphate aldolase	*E. coli* MG1655	U00096	84	97
LC027370	*fbp*	fructose 1,6-bisphosphatase	*E. coli* MG1655	U00096	96	97
AB823563	*fumC*	fumarate hydratase	*Salmonella enterica*	CP007530	81	93
AB823564	*g6pd*	glucose 6-phosphate dehydrogenase	*E. coli* MG1655	U00096	86	97
AB823565	*gap*	glyceraldehyde 3-phosphate dehydrogenase	*E. albertii* KF-1	CP007025	79	91
AB823566	*glk*	glucokinase	*E. coli* MG1655	U00096	80	93
AB823568	*glmM*	phosphoglucosamine mutase	*E. coli* MG1655	U00096	85	96
AB823569	*glmS*	glucosamine-6-phosphate synthase	*E. coli* MG1655	U00096	87	95
AB823570	*glmU*	uridyltransferase/glucosamine-1-phosphate acetyltransferase	*E. coli* MG1655	U00096	83	91
AB823571	*gltA*	type II citrate synthase	*E. coli* MG1655	U00096	86	96
AB823572	*gpmA*	phosphoglyceromutase	*E. coli* MG1655	U00096	88	96
AB823573	*icd*	isocitrate dehydrogenase	*E. coli* MG1655	U00096	88	97
AB823574	*mdh*	malate dehydrogenase	*E. coli* MG1655	U00096	87	97
LC363529	*nagA*	*N*-acetylglucosamine 6-phosphate deacetylase	*E. coli* MG1655	U00096	84	91
AB823577	*pck*	phosphoenolpyruvate carboxykinase	*E. coli* MG1655	U00096	86	93
AB823579	*pfkA*	phosphofructokinase I	*E. coli* MG1655	U00096	87	95
AB823585	*pgk*	phosphoglycerate kinase	*E. coli* MG1655	CP007025	79	90
LC020430	*pta*	phosphotransacetylase	*C. freundii* MTCC1658	EKS56947	98	100
AB823586	*ptsG*	PTS system glucose-specific transporter subunits IIBC	*E. coli* MG1655	U00096	89	97
AB823587	*pyk*	pyruvate kinase	*E. albertii* KF1	CP007025	85	95
AB823588	*sdhB*	succinate dehydrogenase iron-sulfur subunit	*E. coli* MG1655	U00096	87	96
AB823589	*sucA*	2-oxoglutarate dehydrogenase E1 component	*E. coli* MG1655	U00096	89	94
AB823590	*sucB*	dihydrolipoamide succinyltransferase	*E. coli* MG1655	U00096	87	94
AB823591	*sucD*	succinyl-CoA synthetase subunit alpha	*E. coli* MG1655	U00096	88	95
AB823592	*tpiA*	triosephosphate isomerase	*E. coli* MG1655	U00096	90	95
LC018665	*yjcG*	acetate permease	*E. coli* strain ST2747	CP007394	86	96

## Supplementary Materials

The following material is available online at http://www.mdpi.com/2073-4360/10/3/237/s1: Figure S1. SEM images of *Citrobacter freundii* IFO 13545, which is a Gram-negative rod-shaped bacterium that is phylogenetically very close to *Escherichia coli*; Figure S2. Electrophoresis of *Eco*RV-digested total DNA of *C. freundii* IFO 13545 and its *glmS*-disrupted mutant (*ΔglmS*) (a)*;* corresponding Southern blots (b); and restriction maps of the regions around *glmS* in the genomes of IFO 13545 and the disruptant (c). Lane M: 1-kb DNA ladder (size marker); Lane 1: IFO 13545 (wild type); Lane 2: *ΔglmS*; Figure S3. Electrophoresis of *Afl*II-*Eco*RI-*Sph*I-digested total DNA of *C. freundii* IFO 13545 and its *bfpC-*disruptant (*ΔbfpC*) (a); corresponding Southern blots (b); and restriction maps of the regions around *bfpC* in the genomes of IFO 13545 and the disruptant (c). Lane M: λDNA *Hin*dIII digest (size marker); Lane 1: IFO 13545 (wild type); Lane 2: *ΔbfpC*; Table S1: Oligonucleotide primers used in this study.
