# Supplementary material for: Biosynthetic Pathway and Genes of Chitin/Chitosan-Like Bioflocculant in the Genus Citrobacter
Source: Polymers (Basel). 2018 Feb 27;10(3):237. doi: 10.3390/polym10030237 (PMC6414854; doi:10.3390/polym10030237)
Supplement: Supplementary file 1 [file polymers-10-00237-s001.pdf]

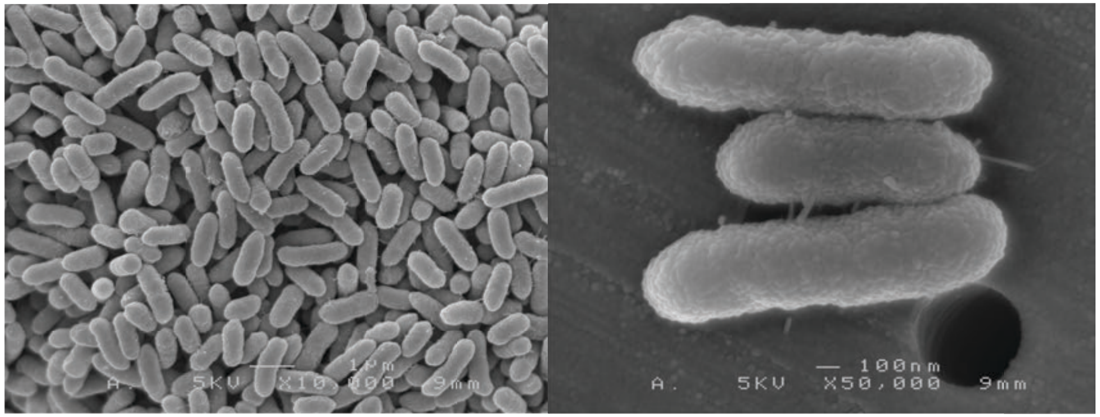

Figure S1. SEM images of *Citrobacter freundii* IFO 13545, which is a Gram-negative rod-shaped bacterium that is phylogenetically very close to *Escherichia coli*.

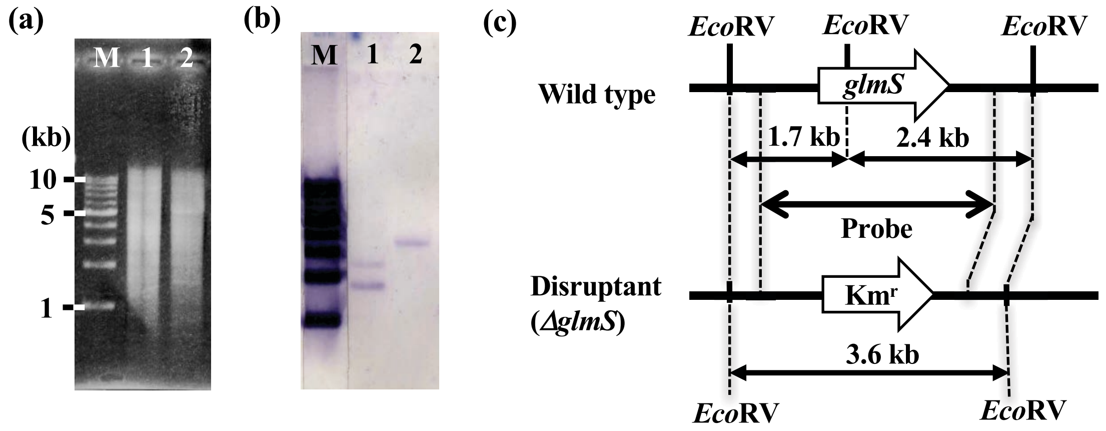

Figure S2. Electrophoresis of *Eco*RV-digested total DNA of *C. freundii* IFO 13545 and its *glmS*-disrupted mutant (Δ*glmS*) (a), corresponding Southern blots (b), and restriction maps of the regions around *glmS* in the genomes of IFO 13545 and the disruptant (c). Lane M: 1-kb DNA ladder (size marker); Lane 1: IFO 13545 (wild type); Lane 2: Δ*glmS*.

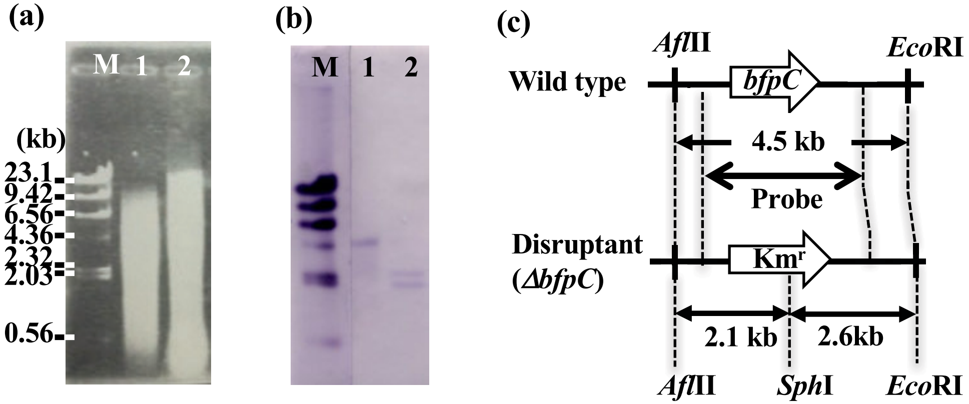

Figure S3. Electrophoresis of *Afl*II-*Eco*RI-*Sph*I-digested total DNA of *C. freundii* IFO 13545 and its *bfpC*-disruptant (*ΔbfpC*) (a), corresponding Southern blots (b), and restriction maps of the regions around *bfpC* in the genomes of IFO 13545 and the disruptant (c). Lane M: λDNA *Hin*dIII digest (size marker); Lane 1: IFO 13545 (wild type); Lane 2: *ΔbfpC*.

Table S1. Oligonucleotide primers used in this study.

Primer name	Sequence
acs-up	F : 5'-GTTCAGCGTGAACTGCAGAG-3'<br>R : 5'-AAGCAGCTCCAGCCTACACAAAATGTAGGGGCTTTGAGAG-3'
acs-down	F : 5'-CTAAGGAGGATATTCATATGTAACTGTGAAATGCCCGATG-3'<br>R : 5'-TGTAGATTCCGGTCAGGATG-3'
aceA-up	F : 5'-CGGCACATGGATTGCTCATC-3'<br>R : 5'-AAGCAGCTCCAGCCTACACAGCAGGGTCAAGAAATCGATC-3'
aceA-down	F : 5'-CTAAGGAGGATATTCATATGTGTAGGCCGGATAAGCGAAC-3'<br>R : 5'-TCGCTAAGGACGCGAGTTAG-3'
glmS-up	F : 5'-TCTGGGTAAAGGCTCCAAAG-3'<br>R : 5'-AAGCAGCTCCAGCCTACACAGACCTGTATTTGCCCATTTC-3'
glmS-down	F : 5'-CTAAGGAGGATATTCATATGTGTTAGCTTGTAGATTGAGC-3'<br>R : 5'-CGTACTGTCAATCACTTCAC-3'
cpsA-up	F : 5'AAGTGACCCGGGTATTCATC3'<br>R: 5'AAGCAGCTCCAGCCTACACACACAAGGGTAGATTGTCAGC3'
cpsA-down	F : 5'CTAAGGAGGATATTCATATGCCGGAATTGTGATATCGCAG3'<br>R: 5'ACAACGCCAGTTCCGCTTTC3'
bfpC-up	F : 5'TTTGAAGATGCGTCACCG-3'<br>R : 5'AAGCAGCTCCAGCCTACACATAAAGAT GACAGCCACGC-3'
bfpC-down	F : 5'-CTAAGGAGGATATTCATATGATGCTTAA ACGCAAACGC-3'<br>R : 5'-TATCCACGACATTGCCTCC-3'
nagA-Km	F : 5'-GGCGGCATTTTTTGAAATCGGGGGTCGGAATGTATGCTTTAACCCAGGGCT GTGTAGGCTGGAGCTGCTT-3'<br>R: 5'-CTCAGTAACGACCTCGTTGCCATTAACGATGGTCTTGATAATTTTAAAGTC A TATGAATATCCTCCTTAG-3'
pkD4-Km	F : 5'-TGTGTAGGCTGGAGCTGCTT-3'<br>R : 5'-CATATGAATATCCTCCTTAG-3'
aceA-RT	F : 5'-TCATATGGGCGGCAAAGTTC-3'<br>R : 5'-TAGAACCCTTCGCTGGTACG-3'
ack-RT	F : 5'-TCTTACCTCTACGCCCTGCC-3'<br>R : 5'-AGACCCATGGAGGTATCAAC-3'
acs-RT	F : 5'-AGCCGAAAGGCGTACTGCAC-3'<br>R : 5'-ACATACGCGCCGGGGTAGGC-3'
bfpC-RT	F : 5'-AATCCGCGTATTCGAACCCG-3'<br>R : 5'-TATCTTCAGTGATCATGTCCG-3'
fba-RT	F : 5'-AGCAGGCGTTCAATATGGGC-3'<br>R : 5'-AACATGGTAATCCACGCCATC-3'
fbp-RT	F : 5'-TTAACGTCTCTGTCGGTACG-3'<br>R : 5'-GTTAATGGAGTACGTGCTGC-3'
glmS-RT	F : 5'-TACAACTCCGGTATGGTGTCCT-3'<br>R : 5'-GCCCAGCTCTTTAGATAAGCG-3'
icd-RT	F : 5'-GCAACATCATGAAGTTCACCG-3'<br>R : 5'-CGCAGCAGGATCTGTTGTAGG-3'
pfkA-RT	F : 5'-CGAAACGCCTGACTGAAATG-3'<br>R : 5'-ACCACTACGAACTCACAACC-3'
pck-RT	F : 5'-AACTGTCTAAAGATGCCGAGC-3'<br>R : 5'-CAGTCAGGAAGATCACTTTGG-3'
ptsG-RT	F : 5'-ATTCAGACGTTCTCCCAGTG-3'<br>R : 5'-AGAGTGCCAGATAGCGATAG-3'
yjcG-RT	F : 5'-CGCTCTGTCGTTAGGTCTTGG-3'<br>R : 5'-AAGCCGATGATAAAGGTCAGG-3'
16S-RT	F : 5'-CAGGCTAGAGTCTTGTAGAG-3'<br>R : 5'-TCGACTTAACGCGTTAGCTC-3'
